# A case report of neonatal alloimmune thrombocytopenic purpura: the importance of correct diagnosis for future pregnancies

**DOI:** 10.1590/S1516-31802005000400008

**Published:** 2005-07-07

**Authors:** Rita Fontão-Wendel, Silvano Wendel, Vicente Odone, Jorge David Carneiro, Leandra Silva, Eduardo Isfer

**Keywords:** Platelet antibody, Alloimmunization, Thrombocytopenia, Immunoglobulin, Human platelet antigens, Aloanticorpos, Alo-antígenos plaquetários, Trombocitopenia, Imunoglobulina G, Antí- genos de plaquetas humanas

## Abstract

**CONTEXT:**

Neonatal alloimmune thrombocytopenic purpura (NAITP) is a neonatal disorder characterized by maternal alloimmunization against fetal platelet antigens inherited from the father. Intracranial hemorrhage leading to death or permanent neurological disability may occur in the fetus.

**CASE REPORT:**

A healthy 30-year-old woman gave birth to her first baby by cesarean after an uneventful 36-week pregnancy. Ten hours after birth, the infant presented severe petechiae, with platelet count of 8 × 10^3^/µl. The mother's platelet count was normal (180 × 10^3^/µl). The infant received intravenous immunoglobulin and was discharged 18 days later, with platelet count of 100 × 10^3^/µl. The cause of thrombocytopenia was not elucidated at that time. One year later, the infant died of neuroblastoma. Since the parents wanted another child, they were referred for investigation of this thrombocytopenia. Platelet genotyping and platelet antibody screening were performed, showing total HPA-1 system mismatch between mother (HPA-1b1b) and father (HPA-1a1a), with anti-HPA-1a antibodies in the mother's serum. We concluded that the first baby was born with NAITP. Thus, in the second pregnancy, the mother was treated with several infusions of intravenous immunoglobulin. Careful ultrasound monitoring was performed, with normal results for mother and fetus throughout the pregnancy. The second baby was born by cesarean at 39 weeks, presenting 92x10^3^ platelets/µl six hours after birth. The baby's platelets were genotyped as HPA-1a1b and the mother's serum again showed anti-HPA-1a antibodies. No clinical bleeding was observed. Intravenous immunoglobulin therapy was an effective treatment for preventing NAITP in the second baby.

## RESUMO

**CONTEXTO:**

Púrpura trombocitopênica neonatal aloimune (PTNA) é uma doença neonatal caracterizada por aloimunização materna contra as plaquetas fetais, que apresentam antígenos herdados do pai. Podem ocorrer hemorragias cerebrais, levando à morte ou a anomalias neurológicas permanentes.

**RELATO DE CASO:**

Mulher saudável, de 30 anos, deu à luz, por parto cesariano na 36^a^ semana de gestação, seu primeiro filho. Com 10 horas de vida, o recém-nascido apresentou petéquias e contagem de 8 × 10^3^ plaquetas/**µl no sangue periférico; foi medicado com imunoglobulina e recebeu alta após 18 dias de** internação, com 100 × 10^3^ plaquetas/**µl. A causa da trombocitopenia não foi elucidada na época. Um ano** depois, a criança morreu de neuroblastoma. Como os pais desejavam outro filho, foram encaminhados para investigação da trombocitopenia. Genotipagem plaquetária e pesquisa de anticorpos antiplaquetários foram realizadas, mostrando total falta de concordância entre os sistemas HPA-1 do pai (HPA-1a1a) e da mãe (HPA-1b1b) e anticorpos anti-HPA-1a no soro da mãe. Concluímos que o primeiro bebê nasceu com PTNA. Por isso, na segunda gravidez, a mãe foi tratada com diversas infusões de imunoglobulina intravenosa. Foi realizado cuidadoso monitoramento por ultra-som, com resultados normais para mãe e feto durante a gravidez. O segundo bebê nasceu por cesárea às 39 semanas, apresentando 92 × 10^3^ plaquetas/µl seis horas após o nascimento. As plaquetas do recém-nascido foram genotipadas como HPA-1a1b e o soro da mãe novamente mostrou anticorpos anti-HPA-1a. Não houve hemorragia. A terapia de infusão de imunoglobulina foi efetiva na prevenção da PTNA no segundo filho.

## INTRODUCTION

Neonatal alloimmune thrombocytopenic purpura (NAITP) is a neonatal disorder characterized by maternal alloimmunization against fetal platelet antigens inherited from the father, which are not present in the mother. Its incidence is estimated to be around one in 350 to one in 2000 live births.^1^

NAITP is considered to be the counterpart of Rh hemolytic disease in newborns, but it involves maternal alloantibodies against platelet antigen systems and may affect the first child (around 30% of cases). Usually, the mother has a healthy and normal pregnancy with a normal platelet count. Nevertheless, severe thrombocytopenia in the fetus or newborn may be observed and intracranial hemorrhage may occur, leading to death or permanent neurological disability.^1^ Therefore, the prenatal management of fetuses at risk is very important. However, there are still a lot of questions about what would be the best treatment.^2^ Some experts have suggested fetal blood sampling, to check the fetal platelet count, but this procedure is very dangerous for the fetus and mother. Others have suggested intravenous injection of immunoglobulin, with or without corticosteroids, for the mother. The diagnosing of NAITP is also difficult as it is initially based on clinical information and depends on ruling out other causes of thrombocytopenia. Laboratory testing is needed to confirm the presence of circulating maternal antibodies against fetal platelets or the presence of platelet antigen incompatibility between father and mother, especially if platelet transfusion is needed to correct the thrombocytopenia. If platelet transfusion is required, it is mandatory to investigate the compatibility between the patient and donor, as these antibodies are clinically important and may cause platelet destruction.

Platelet immunohematology has undergone major development over recent decades, and more sensitive techniques have become available for correctly identifying platelet antibodies and/or platelet antigens.^3^ The most important platelet antigen system involved in NAITP is HPA-1 (human platelet antigen 1), which accounts for 75-85% of cases, followed by HPA-5 (10-20% of cases).^1^ Both are biallelic systems formed by a single nucleotide substitution in the DNA sequence, corresponding to a single amino acid substitution in the respective protein. Usually the **a** allele has a high frequency in Caucasian populations, and the **b** allele has a lower frequency. The phenotype frequency of these antigens in the blood donor population at our institution are: HPA-1a1a: 70.5%; HPA-1a1b: 27.5%; HPA-1b1b: 2.0%; HPA-5a5a: 80.4%; HPA-5a5b: 18.9%; and HPA-5b5b: 0.8% (personal observations).

We report on the case of a mother typed as HPA-1b1b, with anti-HPA-1a antibodies detected in her serum, whose first baby (probably HPA-1a1b) was born with NAITP. Because of correct clinical and laboratory diagnosis of the first baby's condition, successful prenatal management for the second child was possible and no NAITP was observed.

## CASE REPORT

A healthy 30-year-old woman (primipara and primigravida) gave birth to her first baby by cesarean section after an uneventful 36-week pregnancy. Ten hours after birth, the infant presented severe petechiae, with a platelet count of 8 × 10^3^/µl. The mother's platelet count was normal. The infant received intravenous immunoglobulin and was discharged 18 days later with a platelet count of 100 × 10^3^/µl. One year later, the infant died of another complication (neuroblastoma).

Since the parents wanted another child in the near future, it was suggested that immunohematological evaluation of platelets in the family should be undertaken, because of the unexplained thrombocytopenia presented by the first baby. For this purpose, the couple went to our service for platelet genotyping and platelet antibody screening. Whole blood samples were collected from the mother and father for HPA-1, HPA-2, HPA-3, HPA-4, HPA-5 and HPA-6 genotyping. The genomic DNA was extracted using a commercially available DNA isolation kit (DNAzol BD reagent - Life Technologies). HPA genotyping was performed using the polymerase chain reaction technique with sequence specific primers (PCR-SSP), as described by Meyer et al.^4^ Human leukocyte antigen (HLA) typing was also performed in the mother's sample. HLA class I typing was performed by serological tests (microlymphotoxicity) and HLA class II typing by PCR-SSP.

For platelet antibody screening, the mother's serum was tested against a panel of genotyped platelets from known blood donors, using two different techniques: monoclonal antibody immobilization of platelet antigens (MAIPA)^3^ and the platelet immunofluorescence test (PIFT) by flow cytometry.^3^

The mother's HPA genotype was found to be: **HPA-1b1b**; HPA-2a2a; HPA-3a3b; HPA-4a4a; HPA-5a5a; HPA-6a6a. The father's HPA genotype was: **HPA-1a1 a**; HPA-2a2a; HPA-3b3b: HPA-4a4a; HPA-5a5b; HPA-6a6a. The mother's HLA genotype was: HLA: A2, A-; B7, B8, Bw6; DRB1*0301, *1302; DRB3*0101, 0301; DQB1*0201, *0609.

The mother showed complete mismatch with the father in the HPA-1 system (mother HPA-1b1b; father HPA-1a1a) and partial mismatch in HPA-5 (mother HPA-5a5a; father HPA-5a5b). The mother also demonstrated the antigen HLA-DRB3*0101, which has a high prevalence among HPA-1b1b mothers with anti-HPA-1a^5^. The platelet antibody screening by MAIPA (Figure 1a) and PIFT (Figure 1b) showed strong anti-HPA-1a alloantibodies in the mother's serum. No HLA or autoantibodies were detected.

**Figure 1:**
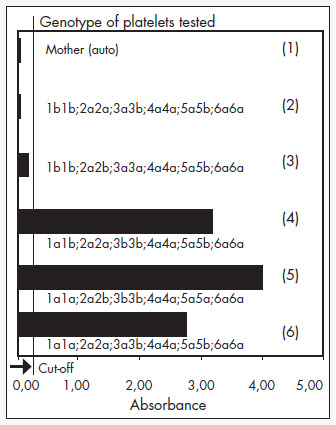
**a)** Absorbance values obtained by MAIPA technique, testing the mother's serum against a panel of genotyped platelets in a case of neonatal alloimune thrombocytopenic purpura. The mother's serum was strongly reactive against HPA-1a1a and HPA-1a1b donor platelets (bars 4, 5, 6) but was negative against HPA-1b1b donor platelets (bars 2, 3), thus showing presence of anti-HPA-1a alloantibodies. No autoantibodies were detected (bar 1).

**Figure 1b):**
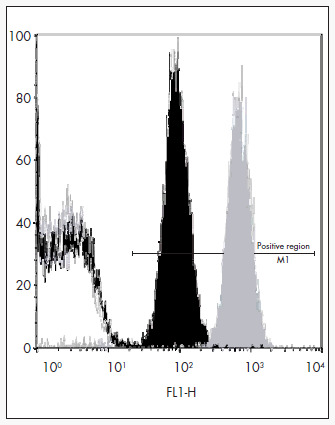
Flow cytometry results. The gray histogram is a positive control; the gray line histogram is a negative control; the positive region is where all the positive histograms must be; the black histogram is the mother's serum against platelets typed HPA-1a1a, showing positive result; the black continuous line (overlapping with negative control) is the mother's serum against platelets typed HPA-1b1b, showing negative result.

Although we had no access to test the baby's platelets (because he died due to neuroblastoma one year after birth), the mother and father's platelet genotyping showed total mismatch in the HPA-1 system, with the presence of anti-HPA-1a in the mother's serum. Since HPA-1 is a biallelic autosomal codominant system, the offspring receives one allele from the father and another from the mother. Because in this case the mother was homozygous for HPA-1b (HPA-1b1b) and the father homozygous for HPA-1a (HPA-1a1a), all their descendents must be HPA-1a1b (heterozygous for HPA-1). From all this evidence, we concluded that the first baby was born with NAITP.

With these findings at hand for the second pregnancy, the mother received intravenous immunoglobulin infusion (400 mg/kg of weight) divided over three consecutive days, starting at the 19^th^ week of gestation. This treatment was repeated at the 24^th^ and 32^nd^ weeks. All ultrasound tests (24^th^, 32^nd^ and 34^th^ weeks) revealed normal fetal evolution. The mother's platelet counts were normal throughout the pregnancy and her second baby was born by cesarean section at the 39^th^ week, with 92 × 10^3^ platelets/µl six hours after birth. The mother's serum again showed anti-HPA-1a antibodies (a titer that was similar to the findings from the first pregnancy) and the baby's platelet genotyping was HPA-1a1b. Although the infant was born with thrombocytopenia (the normal range for platelet counts is 150400 × 10^3^ platelets/µl), no clinical bleeding was observed. Consequently, the mother and child were discharged 72 hours after the birth. The maternal alloimmunization against the infant's platelets was evident, but the intravenous immunoglobulin therapy was an effective treatment for preventing any signs of bleeding in the second baby. Moreover, the fetus was successfully monitored during the pregnancy by ultrasound examinations. The correct diagnosing of the first baby's condition was very important for achieving a good clinical approach for the second child.

Obstetricians should be aware of this disease and the importance of good follow-up for future pregnancies.
